# Singularity analysis of 3-DOF planar parallel continuum robots with constant curvature links

**DOI:** 10.3389/frobt.2022.1082185

**Published:** 2023-01-24

**Authors:** Sven Lilge, Kefei Wen, Jessica Burgner-Kahrs

**Affiliations:** Continuum Robotics Laboratory, Department of Mathematical Computational Sciences, University of Toronto, Mississauga, ON, Canada

**Keywords:** flexible links, parallel robots, continuum robots, kinematics, singularity analysis

## Abstract

This paper presents the singularity analysis of 3-DOF planar parallel continuum robots (PCR) with three identical legs. Each of the legs contains two passive conventional rigid 1-DOF joints and one actuated planar continuum link, which bends with a constant curvature. All possible PCR architectures featuring such legs are enumerated and the kinematic velocity equations are provided for each of them. Afterwards, a singularity analysis is conducted based on the obtained Jacobian matrices, providing a geometrical understanding of singularity occurences. It is shown that while loci and occurrences of type II singularities are mostly analogous to conventional parallel kinematic mechanisms (PKM), type I singularity occurences for the PCR studied in this work are quite different from conventional PKM and less geometrically intuitive. The study provided in this paper can promote further investigations on planar parallel continuum robots, such as structural design and control.

## 1 Introduction

Parallel continuum robots (PCR) are closed loop kinematic mechanisms that make use of continuously bending links (Bryson and Rucker, 2014). They benefit from the inherent compliance, flexibility and lightweight of the continuum links, while the parallel structure can increase their accuracy, payload and operation speed in comparison to conventional continuum robots. Due to their properties, PCR might be utilized in a number of application areas, such as safe human robot collaboration, handling or assembling fragile workpieces as well as industrial inspection and repair in enclosed environments.

Most PCR research to date is concerned with designs that make use of passively deforming continuum links in their structures. Linear or rotary actuators are attached to the proximal end of each link to enable control of a common end-effector. One of such architectures is a 6 degrees-of-freedom (DOF) continuum Stewart-Gough platform (Bryson and Rucker, 2014). Other designs found in the literature include a continuum Delta robot (Yang et al., 2018), which employs flexible continuous joints in combination with rigid links, re-configurable manipulators (Mahoney et al., 2016) as well as structures with additional intermediate constraints (Orekhov et al., 2017; Chen et al., 2018). Rather than using passively deforming continuum links, a number of PCR designs have been proposed in which the deformation of these links is actively controlled. Prominent example of such PCR designs include are pneumatically actuated soft parallel robots (Hopkins et al., 2015; Lindenroth et al., 2019; Garcia et al., 2021), tendon-driven designs (Lilge et al., 2020; Nuelle et al., 2020; Böttcher et al., 2021) or designs utilizing polymer actuators (Moghadam et al., 2015).

One particularly interesting and widely used architecture in the realm of rigid parallel kinematic mechanisms (PKM) are planar 3-DOF manipulators, allowing the end-effector positioning and orientation in a plane (Bonev et al., 2003). Recent research has focused on creating similar mechanisms featuring continuum links (Moghadam et al., 2015; Lilge et al., 2020; Nuelle et al., 2020; Mauzé et al., 2021; Yan et al., 2021; Zaccaria et al., 2022), potentially enabling applications such as micro-positioning, fragile workpiece handling, human-robot collaboration or assembly tasks which are prone to jamming. In this paper, we are extending the current state of the art on planar PCR by further exploring the design space of such structures. We are particularly focusing on 3-DOF designs consisting of three identical kinematic legs, each featuring a continuum link, that is, actively controlled in bending. In our previous work we have investigated two particular designs of such PCR utilizing tendon actuation to bend the continuum links (Lilge et al., 2020; Nuelle et al., 2020; Wen and Burgner-Kahrs, 2022). Both designs were derived from the well known 3-RRR planar PKM by substituting different revolute joints with tendon-driven continuum links. The bending in each continuum link is be described by constant curvature arcs, which in return allows to derive relatively simple and straightforward kinematic models, that solely depend on geometry. Thus, the resulting modeling equations are obtained in a similar form to conventional PKM, allowing to apply methods from this established field to the novel field of PCR.

Throughout the following we are building upon our previous work. Specifically, we are enumerating and investigating all possible planar 3-DOF PCR designs that feature actuated continuum links, whose bending can be described with constant curvature arcs, in addition to two passive joints, which can be revolute or prismatic. Afterwards, the velocity equations for each design are derived using constant curvature assumptions, similar to the approach from Wen and Burgner-Kahrs (2022). Finally, the derived equations are used to investigate and analyze type I and type II singularities in each design. The analysis of such singularities and their conditions is crucial for a number of applications such as robot design, path planning, or control. Related work on singularity analysis for serial continuum robots can for instance be found in (Mayer and Sawodny, 2018; Wang et al., 2021) or (Shihora and Simaan, 2022). Further, Altuzarra and Campa (2020) and Briot and Goldsztejn (2021) investigate the conditions of singularity occurrences for passive link PCR based on a kinetostatic framework. In our work, we make use of our derived kinematic velocity equations to provide geometrical insights and intuitions into the occurrences of type I and type II singularities for the proposed 3-DOF PCR designs, while drawing comparisons to the singularity analysis of rigid 3-DOF planar parallel robots. We note that our paper takes inspiration from Bonev et al. (2003), who enumerate and investigate all possible planar 3-DOF rigid PKM and extend their framework to PCR. We also acknowledge the related work of Merlet (1996), in which all solutions of the forward kinematics for every possible architecture of planar 3-DOF rigid PKM are derived and discussed.

We believe that our study will be useful for additional investigations of the design and control of planar PCR.

## 2 Planar 3-DOF PCR designs

In this section, we are introducing and describing the PCR designs studied throughout this paper. First, the actuated constant curvature links present in each of these designs will be defined. Afterwards, we will enumerate all of the possible planar 3-DOF PCR designs featuring such links while describing the utilized notation. An overview summarizing the nomenclature used in this paper can be found in Table 1.

**TABLE 1:** Nomenclature.

F	Flexible, continuum link	**E**_*i*_	Matrix relating ${\dot{u}}_{i1}$ to $u_{i1}{\dot{\kappa }}_{i}$
P	Prismatic joint	**E**	90° rotation matrix
R	Revolute joint	*A*_*i*_	Reference position of the first rigid joint of continuum link of a leg
*Oxy*	Fixed frame attached to robot’s base	*B*_*i*_	Reference position of the endpoint of a leg
*O*′*x*′*y*′	Moving frame attached to robot’s end-effector	**a**_*i*_	Position vector pointing to *A*_*i*_ expressed in the fixed frame
**p**	Position of moving frame w.r.t. fixed frame	**b**_*i*0_	Position vector pointing to *B*_*i*_ expressed in the moving frame
**Q**	Orientation of moving frame w.r.t. fixed frame	*ϕ*	Rotation angle of the moving frame w.r.t. the fixed frame
*ℓ*_*i*_	Length of *i*th continuum link	**J**	Jacobian matrix relating Cartesian velocities to time derivative of curvatures
*κ*_*i*_	Curvature of *i*th continuum link	**K**	Jacobian matrix relating time derivative of curvatures to Cartesian velocities
*q*_*i*_	Actuation value of *i*th continuum link	**t**	Linear and angular Cartesian velocities
*O*_*i*1_ *x*_*i*1_ *y*_*i*1_	Frame attached to the proximal end of the *i*th continuum link	$\dot{\kappa }$	Vector of the time derivatives of the curvature values
**Q**_*i*1_	Orientation of the *i*th continuum link’s proximal end frame w.r.t. the fixed	**D**	Jacobian relating actuator velocities to time derivative of curvature
	frame		
*O*_*i*2_ *x*_*i*2_ *y*_*i*2_	Frame attached to the distal end of the *i*th continuum link	$\dot{q}$	Vector of actuator velocities
**Q**_*i*2_	Orientation of the *i*th continuum link’s distal end frame w.r.t.	**v**_*i*_	Vector pointing from the start point to the endpoint of the rigid part of a leg
	its proximal end frame		of the rigid part of a leg
**u**_*i*1_, **u**_*i*_	Vector pointing from the proximal to the distal end of the i*th*	**n**_*i*_	Normalized vector orthogonal to **v**_*i*_
	continuum link, expressed in its proximal end frame and		
	the fixed frame, respectively		

### 2.1 Actuated constant curvature link

**FIGURE 1:**
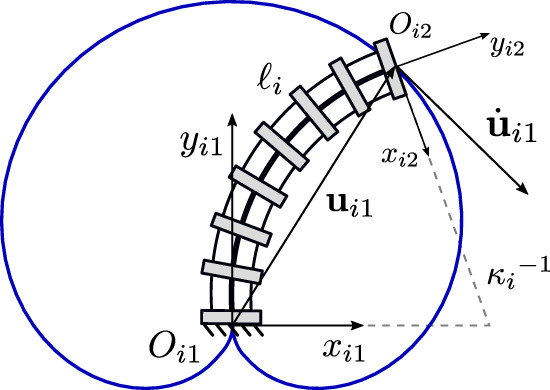
Definition and geometric description of a planar constant curvature link. The trajectory of the link’s distal end is shown in blue.

The continuum links utilized in each of the studied planar PCR designs is shown in Figure 1. Throughout this work, we assume that the length of each link is fixed, while the bending can be actively controlled. We further assume, that bending occurs in arcs with a constant curvature, which is a common assumption for a variety of actuation methods (Webster III and Jones, 2010), e.g., utilizing tendons (Rao et al., 2021), multi-backbones (Simaan et al., 2004), push-pull rods (Oliver-Butler et al., 2021), polymer actuators (Moghadam et al., 2015) or pneumatic actuators (Garcia et al., 2020; 2021). We do not consider additional methods of actuation, such as translating or rotating the bases of the continuum links, a practice often done in PCR with passively deforming links. We note that the continuum links throughout this paper are depicted as tendon-driven structures (i.e. including tendons and tendon routing disks). We do this for illustrative purposes only, without loss of generality of the outlined methodology.

We note that while constant curvature assumptions are generally valid in free space, the tip of the continuum links in our designs may be loaded by the weight of the moving platform and by wrenches arising from the coupling between the individual links and end-effector platform. However, the moving platform can be made lightweight in a practical design, such as done by Nuelle et al. (2020), where a constant curvature based kinematic model has been validated for a planar tendon-driven PCR. Moreover, choosing a constant curvature kinematic description is convenient and intuitive as it allows to compare the kinematic properties of the planar 3-DOF PCR with those of rigid planar 3-DOF parallel manipulators (Lilge et al., 2020; Wen and Burgner-Kahrs, 2022).

Figure 1 shows an example continuum link *i* with fixed length *ℓ*_*i*_ and curvature *κ*_*i*_. A local frame *O*_*i*1_ *x*_*i*1_ *y*_*i*1_ is attached to the base of the link. The *x*_*i*1_-axis is parallel to the spacer disk at the link’s base, while the *y*_*i*1_-axis is orthogonal to it. The trajectory of the link’s distal end is shown in blue. The current position **u**_*i*1_ of the link’s distal end depends on *κ*_*i*_ and is expressed in its local base frame using constant curvature kinematics (Webster III and Jones, 2010; Wen and Burgner-Kahrs, 2022)

$$u_{i1}=\frac{1}{\kappa_{i}}\left[\begin{array}{c} 1-\text{cos}\left(\ell_{i}\kappa_{i}\right)\\ \text{sin}\left(\ell_{i}\kappa_{i}\right) \end{array}\right].$$ (1)

Following this equation, the continuum link is bending in the local positive *x*-direction for *κ*_*i*_ > 0 and in the local negative *x*-direction for *κ*_*i*_ < 0. The continuum link is straight and solely extends along the local *y*-axis. Throughout this paper, we assume that the maximum bending curvature is $|\kappa_{i}|=2\pi \ell_{i}^{-1}$ , in which case the continuum link forms a closed circle.

The velocity of the link’s distal end position can be obtained by differentiating Eq. 1 with respect to time:

$${\dot{u}}_{i1}=E_{i}u_{i1}{\dot{\kappa }}_{i},$$ (2)

where

$$E_{i}=\left[\begin{array}{cc} -\frac{1}{\kappa_{i}} & \ell_{i}\\ \frac{\ell_{i}\text{cos}\left(\ell_{i}\kappa_{i}\right)}{1-\text{cos}\left(\ell_{i}\kappa_{i}\right)} & -\frac{1}{\kappa_{i}} \end{array}\right].$$ (3)

The velocity vector ${\dot{u}}_{i1}$ is tangent to the blue trajectory in Figure 1. In order to describe the orientation of the link’s distal end, an additional frame *O*_*i*2_ *x*_*i*2_ *y*_*i*2_ is attached to its tip. This frame describes the orientation of the link’s distal end with respect to its local base frame *O*_*i*1_ *x*_*i*1_ *y*_*i*1_, where **Q**_*i*2_ is the rotation matrix between these two frames (Wen and Burgner-Kahrs, 2022)

$$Q_{i2}=\left[\begin{array}{cc} \text{cos}\left(-\ell_{i}\kappa_{i}\right) & -\text{sin}\left(-\ell_{i}\kappa_{i}\right)\\ \text{sin}\left(-\ell_{i}\kappa_{i}\right) & \text{cos}\left(-\ell_{i}\kappa_{i}\right) \end{array}\right].$$ (4)

The velocity of this orientation can again be obtained by differentiating Eq. 4 with respect to time

$${\dot{Q}}_{i2}=-{\dot{\kappa }}_{i}\ell_{i}{EQ}_{i2},$$ (5)

where

$$E=\left[\begin{array}{cc} 0 & -1\\ 1 & 0 \end{array}\right].$$ (6)

Both, the linear and angular velocities of the link’s distal end depend on the rate of change in curvature ${\dot{\kappa }}_{i}$ , which itself depends on the actuator velocity ${\dot{q}}_{i}$ . The relationship between ${\dot{\kappa }}_{i}$ and ${\dot{q}}_{i}$ is defined by the utilized actuation principle.

### 2.2 Definition and enumeration of studied PCR designs

Each 3-DOF planar PCR design studied throughout this work consists of three identical legs attached to a moving end-effector platform. Each leg consists of an actuated continuum link in addition to two passive joints, which can either be revolute or prismatic, according to Grübler’s mobility formula (Merlet, 2005). An enumeration of possible planar 3-DOF legs, considering different combinations of the continuum link and passive joints, is shown in Table 2. We are using the following kinematic notation for the planar PCR: R and P respectively stand for a passive revolute joint and a passive prismatic joint, while F stands for an actuated continuum link.

**TABLE 2:** All possible planar 3-DOF legs with one actuated continuum link and two passive rigid joints.

RFR	RRF	RFP	RPF
PFR	PRF	PFP^×^	PPF ^×^
FRR	FRP	FPR	FPP^×^

Since the continuum link in each leg is actuated while the revolute and prismatic joints are passive, there are in total 12 planar 3-DOF legs, which is less than the number (21 in total) of rigid planar 3-DOF legs identified by Bonev et al. (2003). Further, following the investigations outlined by Bonev et al. (2003), some of the listed leg designs will lead to uncontrollable linear velocities, if two or more of these legs are used in a 3-DOF PCR, due to the existence of two passive prismatic joints. There are three of such legs which are marked with a superscript ^×^. Thus, only the remaining nine legs yield planar 3-DOF symmetrical PCR.

**FIGURE 2:**
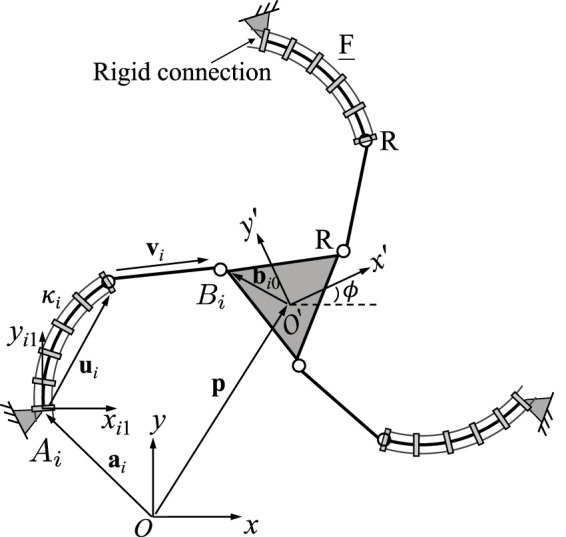
Example design and geometric description of a 3-DOF planar PCR with actuated constant curvature links.

Figure 2 shows the geometric description of a 3-FRR PCR as an example. In order to describe each planar PCR design, a fixed reference frame *Oxy* is attached to its base and a moving frame *O*′*x*′*y*′ is attached to its moving platform. The vector **p** and matrix **Q** respectively represent the position of the origin and the orientation of the moving frame attached to the platform. The rotational angle *ϕ* defines the moving frame’s rotation with respect to the fixed frame. The individual legs of the PCR are denoted with *i* ∈ {1, 2, 3}. The curvature of the *i*th continuum link is described by *κ*_*i*_. The position vector **a**_*i*_ points to a reference point *A*_*i*_ on the first rigid joint or the continuum link of a leg, which could be the centre of the revolute joint (for 3-RFR, 3-RRF, 3-RFP, and 3-RPF PCRs), a point on the axis of the prismatic joint (for 3-PFR and 3-PRF PCRs), or the proximal endpoint of the continuum link (for 3-FRR, 3-FRP and 3-FPR PCRs). Vector **u**_*i*_ is pointing from one end of the continuum link to the other, while vector **v**_*i*_ is pointing from the start point (or the reference point on the prismatic joint) to the endpoint of the rigid part of a leg. Finally, **b**_*i*0_ is the position vector pointing to the endpoint *B*_*i*_ of a leg, expressed in the moving frame *O*′*x*′*y*′. Except for vector **b**_*i*0_, all vectors mentioned above are expressed in the fixed frame *Oxy*.

## 3 Kinematic modeling of planar PCR

Throughout this section, we seek to express the velocity equations of the planar 3-DOF PCR as

$$Jt=K\dot{\kappa },$$ (7)

where **J** and **K** are the Jacobian matrices, **t** is the vector of both the linear and angular Cartesian velocities of the end-effector platform, and $\dot{\kappa }$ is the vector that contains the time derivative of the curvature of the continuum links. For the designs studied throughout this work, **K** will result in a diagonal matrix with $K=\text{diag}\left[\begin{array}{ccc} k_{1} & k_{2} & k_{3} \end{array}\right]$ . Both matrices are obtained through the definition of constraint equations which describe the corresponding PCR designs.

In the following, we are providing detailed derivations of the velocity equations for each of the planar 3-DOF PCR. This includes analytical expressions for the Jacobian matrices **J** and **K**, which are later investigated to identify both type I and type II singularities of the designs. Specifically, type I singularities occur, when **K** becomes singular, and type II singularities occur, when **J** becomes singular. Jacobian matrix **J** is derived in a way, such that the resulting formulations consists of individual Plücker lines, which will be useful when investigating its singularity occurrences. Throughout these derivations, we are assuming that the constant curvatures ***κ*** are directly controlled, e.g. using tendons, multi-backbone structures of push-pull rods. Thus, all derivations are stated with respect to the time derivative of these curvatures $\dot{\kappa }$ . Using the particular kinematics of the employed actuation methods, $\dot{\kappa }$ can be related to the joint velocities $\dot{q}$ using the mapping

$$\dot{\kappa }=D\dot{q},$$ (8)

where **D** is a diagonal matrix, with each entry relating the velocity of actuated joint *q*_*i*_ to the change in curvature *κ*_*i*_. In the most general case, matrix **D** is constant and independent from the current curvature of a continuum link. For example, for tendon-driven or multi-backbone designs the entries of **D** are defined by the distance *d*_*t*_ between the routed tendons or secondary backbones and the central structure of a link (Simaan et al., 2004; Rao et al., 2021), such that

$$\left[\begin{array}{c} {\dot{\kappa }}_{1}\\ {\dot{\kappa }}_{2}\\ {\dot{\kappa }}_{3} \end{array}\right]=\left[\begin{array}{ccc} d_{t} & 0 & 0\\ 0 & d_{t} & 0\\ 0 & 0 & d_{t} \end{array}\right]\left[\begin{array}{c} {\dot{q}}_{1}\\ {\dot{q}}_{2}\\ {\dot{q}}_{3} \end{array}\right],$$ (9)

where *q*_*i*_ is the change in tendon or secondary backbone length w.r.t. their initial lengths. It is apparent, that in this simple case, matrix **D** is singularity free at all times. Depending on the robotic prototype, the entries in **D** might also depend on the curvatures ***κ***, which can potentially lead to better kinematic modeling accuracies (Nuelle et al., 2020). However, such a curvature dependent mapping has no impact on the singularity loci, as long as **D** does not become singular itself. Otherwise, potential singularities in **D** would lead to additional type I singularities, as there would be non-zero actuator velocities $\dot{q}$ , leading to curvature time derivatives $\dot{\kappa }$ and consequentially to end-effector velocities **t** that are both equal to zero, effectively loosing a controllable degree of freedom at the platform. However, for non-singular matrices **D**, which is a reasonable assumption for the planar designs considered throughout this work, the singularity loci solely depend on the geometry of the resulting constant curvature arcs, which justifies outlining our derivations with respect to $\dot{\kappa }$ .

We note that such a separation of the constant curvature kinematics of continuum robots into a robot-independent mapping, relating task space variables to arc parameters, and a robot-dependent mapping, relating actuation variables to arc parameters, is a common practice (Webster III and Jones, 2010). In our case, matrix **K** describes the robot-independent velocity mapping and matrix **D** the robot-dependent one.

### 3.1 3-RFR PCR

**FIGURE 3:**
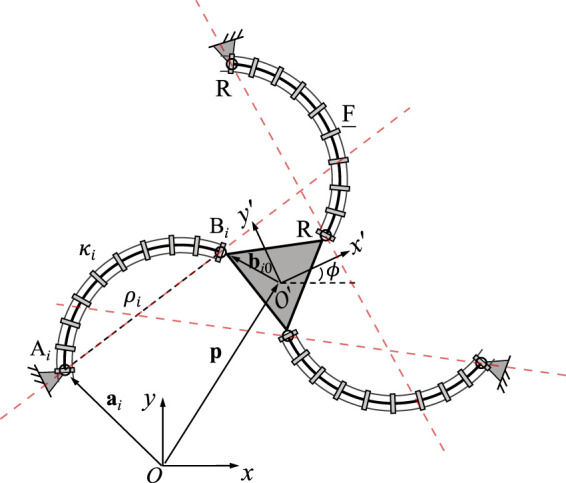
Geometric description of a planar three-DOF 3-RFR PCR.

A planar 3-RFR PCR is illustrated in Figure 3, where *ρ*_*i*_ stands for the distance between points *A*_*i*_ and *B*_*i*_. Kinematic modeling of this architecture has been developed in (Lilge et al., 2020; Wen and Burgner-Kahrs, 2022), hence only the expressions of the Jacobian matrices are given here for the sake of clarity. Jacobian matrix **J** of the PCR is written as

$$J=\left[\begin{array}{cc} u_{1}^{T} & u_{1}^{T}{EQb}_{10}\\ u_{2}^{T} & u_{2}^{T}{EQb}_{20}\\ u_{3}^{T} & u_{3}^{T}{EQb}_{30} \end{array}\right],$$ (10)

where

$$u_{i}=p+{Qb}_{i0}-a_{i}.$$ (11)

Here, **p** and **Q** are the position and orientation of the moving frame *O*′*x*′*y*′ with respect to the fixed frame *Oxy*, respectively. Thus, **u**_*i*_ is pointing from *A*_*i*_ to *B*_*i*_. The rotation matrix **E** is defined in Eq. 6. Jacobian matrix **K** can be written as

$$K=\text{diag}\left[\begin{array}{ccc} k_{1} & k_{2} & k_{3} \end{array}\right],$$ (12)

where

$$k_{i}=\rho_{i}\alpha_{i},$$ (13)

with

$$\rho_{i}=\frac{2}{\kappa_{i}}\text{sin}\left(\frac{\ell_{i}\kappa_{i}}{2}\right),\;\alpha_{i}=\frac{\ell_{i}}{\kappa_{i}}\text{cos}\left(\frac{\ell_{i}\kappa_{i}}{2}\right)-\frac{\rho_{i}}{\kappa_{i}}.$$ (14)

The derivation of these equations can be found in (Wen and Burgner-Kahrs, 2022). Each row in Jacobian matrix **J** represents a Plücker line passing through points *A*_*i*_ and *B*_*i*_ in the corresponding leg, as indicated by the dashed red lines in Figure 3. This is similar to the case of the rigid planar 3-RRR parallel manipulator studied by Bonev et al. (2003).

### 3.2 3-FRR PCR

**FIGURE 4:**
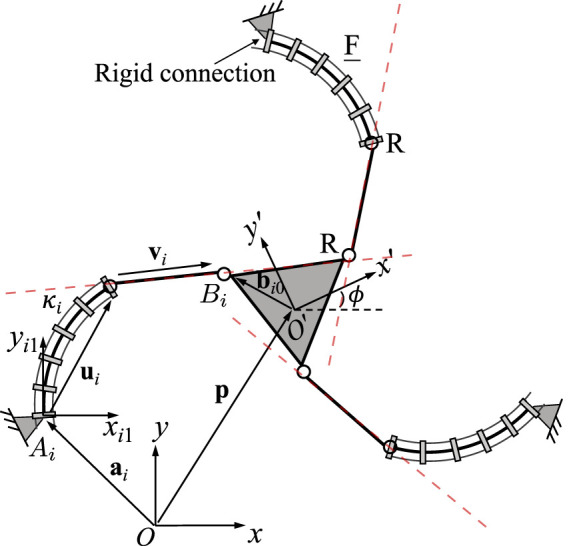
Geometric description of a planar three-DOF 3-FRR PCR.

A planar 3-FRR PCR is illustrated in Figure 4. Again, the kinematic modeling of this architecture has previously been developed in (Lilge et al., 2020; Wen and Burgner-Kahrs, 2022). The Jacobian matrix **J** of the PCR is written as

$$J=\left[\begin{array}{cc} v_{1}^{T} & v_{1}^{T}E{Qb}_{10}\\ v_{2}^{T} & v_{2}^{T}E{Qb}_{20}\\ v_{3}^{T} & v_{3}^{T}E{Qb}_{30} \end{array}\right],$$ (15)

where

$$v_{i}=p+{Qb}_{i0}-u_{i}-a_{i},$$ (16)

and **u**_*i*_ is **u**_*i*1_, as defined in Eq. 1, expressed in the fixed frame *Oxy*. Here, each row represents a Plücker line passing through the rigid link in the corresponding leg, as indicated by the red dashed lines in Figure 4. This is similar to the case of the rigid planar 3-RRR parallel manipulator studied by Bonev et al. (2003). Jacobian matrix **K** can be written as

$$K=\text{diag}\left[\begin{array}{ccc} k_{1} & k_{2} & k_{3} \end{array}\right],$$ (17)

with

$$k_{i}=v_{i}^{T}Q_{i1}E_{i}u_{i1}.$$ (18)

Here, **Q**_*i*1_ is the rotation matrix of the local frame *O*_*i*1_ *x*_*i*1_ *y*_*i*1_, which is attached to the base of the *i*th continuum link, with respect to the fixed frame *Oxy*.

### 3.3 3-RRF PCR

**FIGURE 5:**
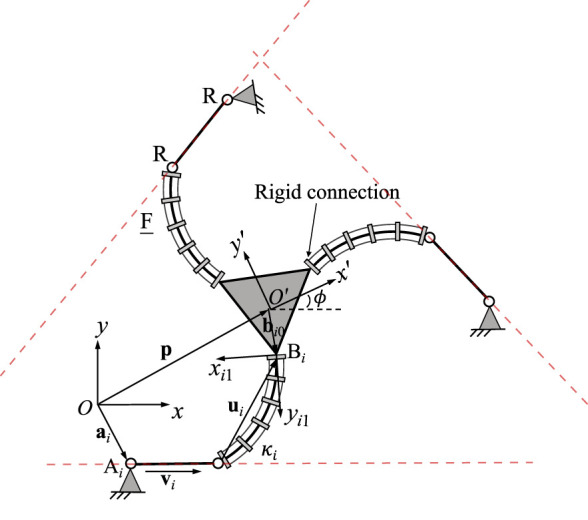
Geometric description of a planar three-DOF 3-RRF PCR.

The geometric description of the 3-RRF PCR is shown in Figure 5. The geometric constraint for each of the legs is that the length of the rigid RR link is constant at any instant, resulting in the following constraint equation

$${\left(p+{Qb}_{i0}-a_{i}-u_{i}\right)}^{T}\left(p+{Qb}_{i0}-a_{i}-u_{i}\right)=v_{i}^{2},$$ (19)

in which *v*_*i*_ is the Euclidean norm of vector **v**_*i*_ connecting the centre of the first revolute joint to that of the second revolute joint in the *i*th leg. Differentiating Eq. 19 with respect to time yields

$$v_{i}^{T}\dot{p}+\dot{\phi }v_{i}^{T}{EQb}_{i0}-v_{i}^{T}{\dot{u}}_{i}=0,$$ (20)

where

$$v_{i}=p+{Qb}_{i0}-a_{i}-u_{i}.$$ (21)

Vector **u**_*i*_ can be expressed in the local frame *O*_*i*1_ *x*_*i*1_ *y*_*i*1_ of continuum link *i*, which is attached to its end connected to the moving platform. The orientation of this local frame with respect to the moving platform frame is constant. We rewrite vector **u**_*i*_ in the following form

$$u_{i}=-Q_{i1}u_{i1},$$ (22)

where **Q**_*i*1_ is the rotation matrix of the local frame with respect to the fixed frame *Oxy*. Vector **u**_*i*1_ is defined in Eq. 1. Differentiating Eq. 22 with respect to time, one can obtain

$${\dot{u}}_{i}=-\dot{\phi }{EQ}_{i1}u_{i1}-Q_{i1}{\dot{u}}_{i1}=\dot{\phi }{Eu}_{i}-Q_{i1}{\dot{u}}_{i1},$$ (23)

in which ${\dot{Q}}_{i1}=\dot{\phi }{EQ}_{i1}$ , and ${\dot{u}}_{i1}$ is defined in Eq. 2. Substituting Eq. 23 into Eq. 20 and rearranging terms leads to

$$v_{i}^{T}\dot{p}+\dot{\phi }v_{i}^{T}E\left({Qb}_{i0}-u_{i}\right)=-{\dot{\kappa }}_{i}v_{i}^{T}Q_{i1}E_{i}u_{i1}.$$ (24)

Jacobian matrices **J** and **K** of the 3-RRF PCR can then be constructed from Eq. 24. Matrix **J** is written as

$$J=\left[\begin{array}{cc} v_{1}^{T} & v_{1}^{T}E\left({Qb}_{10}-u_{1}\right)\\ v_{2}^{T} & v_{2}^{T}E\left({Qb}_{20}-u_{2}\right)\\ v_{3}^{T} & v_{3}^{T}E\left({Qb}_{30}-u_{3}\right) \end{array}\right],$$ (25)

in which each row represents a Plücker line passing through the rigid link in the corresponding leg, as indicated by the red dashed lines in Figure 5, and the diagonal elements in matrix **K** are

$$k_{i}=-v_{i}^{T}Q_{i1}E_{i}u_{i1}.$$ (26)

### 3.4 3-RFP PCR

**FIGURE 6:**
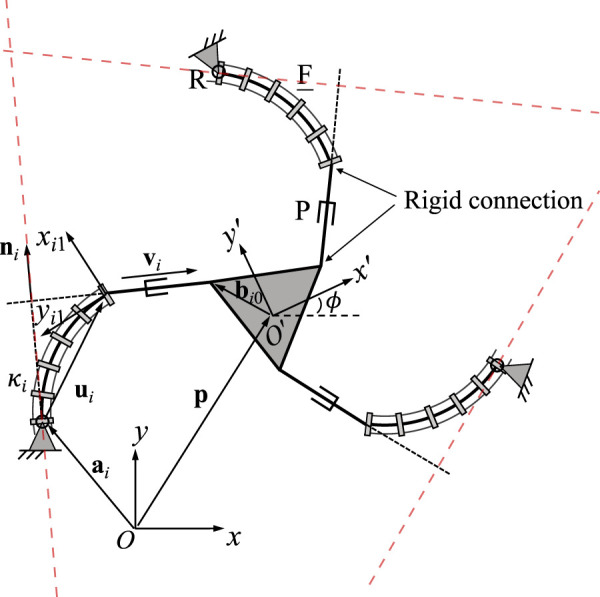
Geometric description of a planar three-DOF 3-RFP PCR.

A planar 3-RFP PCR is schematically represented in Figure 6. The proximal end of the passive prismatic joint in each leg is rigidly connected to the continuum link, while the distal end of this joint is fixed on the moving platform. In order to establish the constraint equations, we first define a unit vector **n**_*i*_ which is expressed in the fixed frame *Oxy* and is orthogonal to vector **v**_*i*_ in the same leg. This vector is constant since the direction of vector **v**_*i*_ with respect to the moving frame is maintained at any instant. The orthogonality between vectors **n**_*i*_ and **v**_*i*_ in each leg can be written as

$$n_{i}^{T}v_{i}=0,$$ (27)

where vector **v**_*i*_ is expressed in the same form as that shown in Eq. 21. To make the following derivations more intuitive, we rewrite the constraint equation as

$${\left({Qn}_{i0}\right)}^{T}v_{i}=0,$$ (28)

where *n*_*i*0_ is the unit vector **n**_*i*_ expressed in the moving frame *O*′*x*′*y*′. The time derivative of Eq. 28 yields

$$\dot{\phi }{\left({EQn}_{i0}\right)}^{T}v_{i}+{\left({Qn}_{i0}\right)}^{T}{\dot{v}}_{i}=0,$$ (29)

with

$${\dot{v}}_{i}=\dot{p}+\dot{\phi }{EQb}_{i0}-{\dot{u}}_{i}.$$ (30)

Similarly to the local frame defined in Figure 5, a local frame *O*_*i*1_ *x*_*i*1_ *y*_*i*1_ is attached to the distal end of the *i*th continuum link of the 3-RFP PCR (see Figure 6). The orientation matrix of this frame relative to the fixed frame is denoted by **Q**_*i*1_, which is a function of angle *ϕ*, i.e., the rotational angle of the moving frame with respect to the fixed frame. This is because the orientation of this local frame relative to the moving platform frame is constant, regardless of the prismatic joint values in the same leg. The time derivative of vector **u**_*i*_ can be derived in the same way as in the previous design and results in Eq. 23.

Substituting the expression of ${\dot{u}}_{i}$ into Eq. 30 and then into Eq. 29 and noting that **E** ^*T*^ = −**E**, the left summand of Eq. 29, which contains $\dot{\phi }$ , can be rewritten as

$$\begin{array}{ll} & \;\dot{\phi }{\left({EQn}_{i0}\right)}^{T}v_{i}+\dot{\phi }{\left({Qn}_{i0}\right)}^{T}\left({EQb}_{i0}\right)-\dot{\phi }{\left({Qn}_{i0}\right)}^{T}\left({Eu}_{i}\right)\\ & \;=\dot{\phi }{\left({Qn}_{i0}\right)}^{T}E\left({Qb}_{i0}-v_{i}-u_{i}\right)\\ & \;=\dot{\phi }h_{i}, \end{array}$$ (31)

in which

$$h_{i}={\left({Qn}_{i0}\right)}^{T}E\left({Qb}_{i0}-v_{i}-u_{i}\right).$$ (32)

Hence Eq. 29 can be rewritten into the following form

$${\left({Qn}_{i0}\right)}^{T}\dot{p}+\dot{\phi }h_{i}=-{\dot{\kappa }}_{i}{\left({Qn}_{i0}\right)}^{T}Q_{i1}E_{i}u_{i1}.$$ (33)

The Jacobian matrices **J** and **K** are constructed from Eq. 33, where matrix **J** is written as

$$J=\left[\begin{array}{cc} {\left(Qn_{10}\right)}^{T} & h_{1}\\ {\left(Qn_{20}\right)}^{T} & h_{2}\\ {\left(Qn_{30}\right)}^{T} & h_{3} \end{array}\right]=\left[\begin{array}{cc} n_{1}^{T} & n_{1}^{T}E\left(a_{1}-p_{1}\right)\\ n_{2}^{T} & n_{2}^{T}E\left(a_{2}-p_{2}\right)\\ n_{3}^{T} & n_{3}^{T}E\left(a_{3}-p_{3}\right) \end{array}\right],$$ (34)

in which each row represents a Plücker line which is orthogonal to vector **v**_*i*_ and passes through the centre of the revolute joint in the *i*th leg, as indicated by the red dashed lines in Figure 6. The diagonal elements of matrix **K** are

$$k_{i}=-{\left(Qn_{i0}\right)}^{T}Q_{i1}E_{i}u_{i1}=-n_{i}^{T}Q_{i1}E_{i}u_{i1}.$$ (35)

It should be noted that **n**_*i*_ becomes undefined, when **v**_*i*_ becomes a zero vector. Thus, type I singularities occur in this case, in addition to all other cases in which *k*_*i*_ = 0 (for *i* = 1, 2 or 3). The same is true for the other following planar PCR design which contain passive prismatic joints.

### 3.5 3-PFR PCR

**FIGURE 7:**
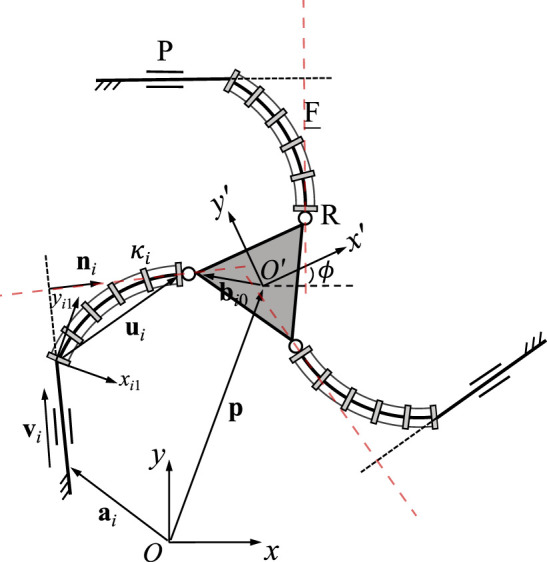
Geometric description of a planar three-DOF 3-PFR PCR.

A planar 3-PFR PCR is schematically represented in Figure 7. The constraint equations of the planar 3-PFR PCR are written as

$$n_{i}^{T}v_{i}=0,$$ (36)

where **n**_*i*_ is a unit vector expressed in the fixed frame which is orthogonal to vector **v**_*i*_. Vector **n**_*i*_ is constant with respect to time, as its direction does not change regardless of the value of the prismatic joint in the same leg. Vector **v**_*i*_ is expressed in the same form as that shown in (21). Vector **u**_*i*_, which is used to express vector **v**_*i*_, is defined as **u**_*i*_ = **Q**_*i*1_ **u**_*i*1_, where **Q**_*i*1_ stands for the constant orientation matrix of the local frame *O*_*i*1_ *x*_*i*1_ *y*_*i*1_ relative to the fixed frame, and vector **u**_*i*1_ is expressed in the local frame *O*_*i*1_ *x*_*i*1_ *y*_*i*1_ using Eq. 1.

The time derivative of Eq. 36 leads to

$$n_{i}^{T}{\dot{v}}_{i}=n_{i}^{T}\left({\dot{p}}_{i}+\dot{\phi }{EQb}_{i0}-Q_{i1}{\dot{u}}_{i1}\right)=0.$$ (37)

Using Eq. 2, we can further write

$$n_{i}^{T}\left(\dot{p}+\dot{\phi }{EQb}_{i0}-{\dot{\kappa }}_{i}Q_{i1}E_{i}u_{i1}\right)=0,$$ (38)

$$n_{i}^{T}\left(\dot{p}+\dot{\phi }{EQb}_{i0}\right)={\dot{\kappa }}_{i}n_{i}^{T}Q_{i1}E_{i}u_{i1},$$ (39)

where **E**_*i*_ is defined in Eq. 3. Using Eq. 39, Jacobian matrix **J** can be written in the following form

$$J=\left[\begin{array}{cc} n_{1}^{T} & n_{1}^{T}{EQb}_{10}\\ n_{2}^{T} & n_{2}^{T}{EQb}_{20}\\ n_{3}^{T} & n_{3}^{T}{EQb}_{30} \end{array}\right].$$ (40)

The Plücker line in each leg is orthogonal to vector **v**_*i*_ and passes through the centre of the revolute joint, as indicated by the red dashed lines in Figure 7. The diagonal elements in Jacobian matrix **K** are

$$k_{i}=n_{i}^{T}Q_{i1}E_{i}u_{i1}.$$ (41)

### 3.6 3-PRF PCR

**FIGURE 8:**
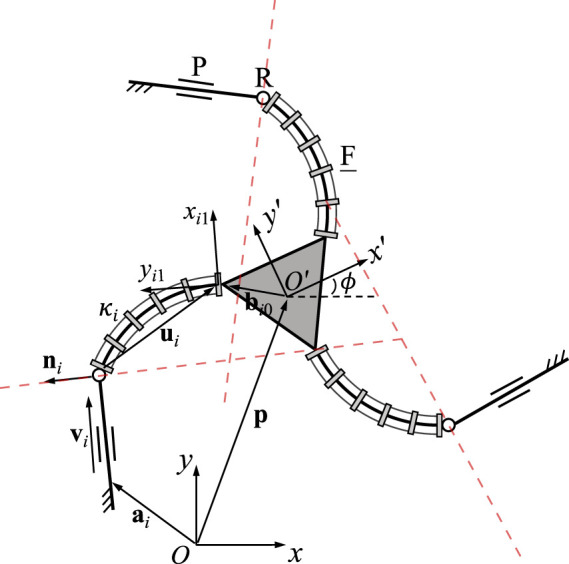
Geometric description of a planar three-DOF 3-PRF PCR.

A planar 3-PRF PCR is schematically represented in Figure 8. The derivation is similar to the one of the previous design, thus the constraint equations are

$$n_{i}^{T}v_{i}=0,$$ (42)

where **n**_*i*_ is a unit vector and **v**_*i*_ is defined in Eq. 21. This time, vector **u**_*i*_ is defined as **u**_*i*_ = −**Q**_*i*1_ **u**_*i*1_, where **Q**_*i*1_ stands for local frame *O*_*i*1_ *x*_*i*1_ *y*_*i*1_ relative to the fixed frame, and vector **u**_*i*1_ is expressed in the local frame *O*_*i*1_ *x*_*i*1_ *y*_*i*1_ using Eq. 1. We note that **Q**_*i*1_ is not constant with respect to time here, as it moves with the moving platform. However, the direction of **n**_*i*_ does not change with respect to time and is constant for any joint value of the prismatic joint. In total, the time derivative of the constraint equation is obtained as

$$n_{i}^{T}\left(\dot{p}+\dot{\phi }{EQb}_{i0}-\dot{\phi }{Eu}_{i}+{\dot{\kappa }}_{i}Q_{i1}E_{i}u_{i1}\right)=0,$$ (43)

again making use of Eq. 23 and **E**_*i*_ is again defined in Eq. 3. Based on Eq. 43, we can identify

$$J=\left[\begin{array}{cc} n_{1}^{T} & n_{1}^{T}E\left({Qb}_{10}-u_{1}\right)\\ n_{2}^{T} & n_{2}^{T}E\left({Qb}_{20}-u_{2}\right)\\ n_{3}^{T} & n_{3}^{T}E\left({Qb}_{30}-u_{3}\right) \end{array}\right].$$ (44)

The Plücker line in each leg is orthogonal to vector **v**_*i*_ and passes through the centre of the revolute joint, as indicated by the red dashed lines in Figure 8. The diagonal elements of Jacobian matrix **K** result in

$$k_{i}=-n_{i}^{T}Q_{i1}E_{i}u_{i1}.$$ (45)

### 3.7 3-FRP PCR

**FIGURE 9:**
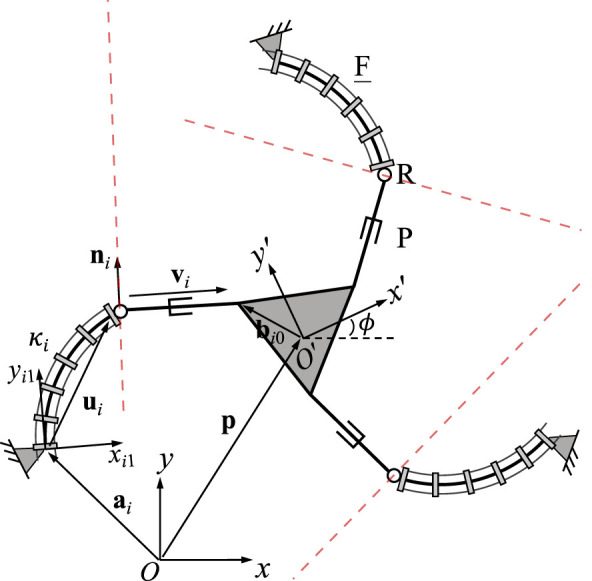
Geometric description of a planar three-DOF 3-FRP PCR.

Similar to the previously discussed designs that feature passive prismatic joints, it can be readily observed that the constraint equations of the planar 3-FRP PCR (see Figure 9) are the orthogonality between the unit vector **n**_*i*_ and vector **v**_*i*_ such that $n_{i}^{T}v_{i}=0$ . Again, **v**_*i*_ is defined in Eq. 21, in which vector **u**_*i*_ is defined as **u**_*i*_ = **Q**_*i*1_ **u**_*i*1_, where **Q**_*i*1_ stands for local frame *O*_*i*1_ *x*_*i*1_ *y*_*i*1_ relative to the fixed frame, and vector **u**_*i*1_ is expressed in the local frame *O*_*i*1_ *x*_*i*1_ *y*_*i*1_ using Eq. 1. Skipping some steps, which have been outlined throughout the previous designs, the time derivation of the constraint equations results in

$${\dot{n}}_{i}^{T}v_{i}+n_{i}^{T}{\dot{v}}_{i}=0,$$ (46)

$$\dot{\phi }{\left({En}_{i}\right)}^{T}v_{i}+n_{i}^{T}\left(\dot{p}+\dot{\phi }{EQb}_{i0}-{\dot{\kappa }}_{i}Q_{i1}E_{i}u_{i1}\right)=0,$$ (47)

$$n_{i}^{T}\dot{p}+\dot{\phi }n_{i}^{T}E\left({Qb}_{i0}-v_{i}\right)={\dot{\kappa }}_{i}n_{i}^{T}Q_{i1}\left.E_{i}u_{i1}\right),$$ (48)

where **E**_*i*_ is again defined in and Eq. 3. Now the Jacobian matrix **J** can be obtained as

$$J=\left[\begin{array}{cc} n_{1}^{T} & n_{1}^{T}E\left({Qb}_{10}-v_{1}\right)\\ n_{2}^{T} & n_{2}^{T}E\left({Qb}_{20}-v_{2}\right)\\ n_{3}^{T} & n_{3}^{T}E\left({Qb}_{30}-v_{3}\right) \end{array}\right].$$ (49)

The Plücker line in each leg is orthogonal to vector **v**_*i*_ and passes through the centre of the revolute joint, as indicated by the red dashed lines in Figure 9. The diagonal elements of the Jacobian matrix **K** consist of

$$k_{i}=n_{i}^{T}Q_{i1}E_{i}u_{i1}.$$ (50)

### 3.8 3-RPF PCR

**FIGURE 10:**
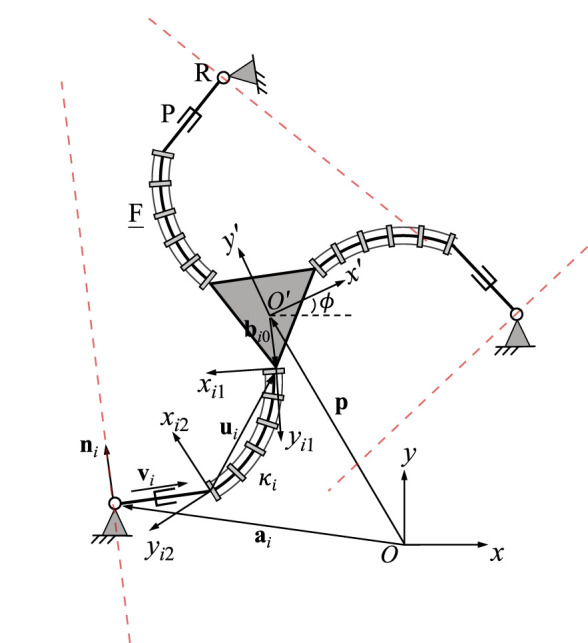
Geometric description of a planar three-DOF 3-RPF PCR.

A planar 3-RPF PCR is schematically represented in Figure 10. Two local frames, noted *O*_*i*1_ *x*_*i*1_ *y*_*i*1_ and *O*_*i*2_ *x*_*i*2_ *y*_*i*2_, are respectively attached to the distal and proximal ends of the continuum links. The orientation of the former local frame relative to the moving platform frame is constant. In each leg, the unit vector **n**_*i*_, which is expressed in the moving platform frame, is orthogonal to vector **v**_*i*_.

Similar to the previously discussed designs, the constraint equations are

$$n_{i}^{T}v_{i}=0,$$ (51)

where **v**_*i*_ is the same as in Eq. 21 and **u**_*i*_ is defined as

$$u_{i}=-Q_{i1}u_{i1},$$ (52)

in which **Q**_*i*1_ is the constant orientation matrix of the local frame *O*_*i*1_ *x*_*i*1_ *y*_*i*1_ with respect to the fixed frame, and **u**_*i*1_ is defined in the local frame *O*_*i*1_ *x*_*i*1_ *y*_*i*1_ as expressed in Eq. 1. The time derivative of the constraint equations yields

$${\dot{n}}_{i}^{T}v_{i}+n_{i}^{T}{\dot{v}}_{i}=0,$$ (53)

with

$${\dot{v}}_{i}=\dot{p}+\dot{\phi }{EQb}_{i0}-{\dot{u}}_{i}=\dot{p}+\dot{\phi }{EQb}_{i0}-\dot{\phi }{Eu}_{i}+{\dot{\kappa }}_{i}Q_{i1}E_{i}u_{i1},$$ (54)

where the expression for ${\dot{u}}_{i}$ can be derived based on the procedure given in Eq. 22 and Eq. 23, **E**_*i*_ has been defined in Eq. 3. In order to calculate the time derivative of vector **n**_*i*_, we rewrite this vector in the following form

$$n_{i}=Q_{i1}Q_{i2}n_{i2},$$ (55)

where **Q**_*i*2_ is the orientation matrix of the local frame *O*_*i*2_ *x*_*i*2_ *y*_*i*2_ relative to the local frame *O*_*i*1_ *x*_*i*1_ *y*_*i*1_ as defined in Eq. 4. Unit vector **n**_*i*2_ denotes unit vector **n**_*i*_ when expressed in the local frame *O*_*i*2_ *x*_*i*2_ *y*_*i*2_. The direction of the unit vector **n**_*i*2_ is constant with respect to time since the geometric relationship between the prismatic joint and the proximal end of the continuum link in the same leg remains unchanged. The time derivative of vector **n**_*i*_ can then be written as

$${\dot{n}}_{i}={\dot{Q}}_{i1}Q_{i2}n_{i2}+Q_{i1}{\dot{Q}}_{i2}n_{i2}.$$ (56)

Since the local frame is attached to the moving frame, its derivation with respect to time depends on the angular velocity of the moving platform such that ${\dot{Q}}_{i1}=\dot{\phi }{EQ}_{i1}$ . Together with the time derivative ${\dot{Q}}_{i2}$ from Eq. 5, we obtain.

$${\dot{n}}_{i}=\dot{\phi }{EQ}_{i1}Q_{i2}n_{i2}-{\dot{\kappa }}_{i}\ell_{i}Q_{i1}{EQ}_{i2}n_{i2}$$ (57)

$$\;\;\;\;=\dot{\phi }{En}_{i}-{\dot{\kappa }}_{i}\ell_{i}En_{i}.$$ (58)

Substituting Eq. 54 and Eq. 58 into Eq. 53 and rearranging terms, and noting that **E** ^*T*^ = −**E** one can obtain.

$$n_{i}^{T}\dot{p}+\dot{\phi }n_{i}^{T}{EQb}_{i0}+\dot{\phi }{\left({En}_{i}\right)}^{T}v_{i}-\dot{\phi }n_{i}^{T}{Eu}_{i}={\dot{\kappa }}_{i}k_{i},$$ (59)

$$n_{i}^{T}\dot{p}+\dot{\phi }n_{i}^{T}{EQb}_{i0}-\dot{\phi }n_{i}^{T}Ev_{i}-\dot{\phi }n_{i}^{T}{Eu}_{i}={\dot{\kappa }}_{i}k_{i},$$ (60)

$$n_{i}^{T}\dot{p}+\dot{\phi }n_{i}^{T}E\left({Qb}_{i0}-v_{i}-u_{i}\right)={\dot{\kappa }}_{i}k_{i},$$ (61)

where

$$k_{i}=-n_{i}^{T}Q_{i1}E_{i}u_{i1}+\ell_{i}{\left(En_{i}\right)}^{T}v_{i},$$ (62)

$$=-n_{i}^{T}Q_{i1}E_{i}u_{i1}-\ell_{i}n_{i}^{T}Ev_{i},$$ (63)

$$=-n_{i}^{T}\left(Q_{i1}E_{i}u_{i1}+\ell_{i}Ev_{i}\right).$$ (64)

The Jacobian matrices now result from Eq. 61 in

$$J=\left[\begin{array}{cc} n_{1}^{T} & n_{1}^{T}E\left({Qb}_{10}-v_{1}-u_{1}\right)\\ n_{2}^{T} & n_{2}^{T}E\left({Qb}_{20}-v_{2}-u_{2}\right)\\ n_{3}^{T} & n_{3}^{T}E\left({Qb}_{30}-v_{3}-u_{3}\right) \end{array}\right]=\left[\begin{array}{cc} n_{1}^{T} & n_{1}^{T}E\left(a_{1}-p\right)\\ n_{2}^{T} & n_{2}^{T}E\left(a_{2}-p\right)\\ n_{3}^{T} & n_{3}^{T}E\left(a_{3}-p\right) \end{array}\right],$$ (65)

and **K** = diag[*k*_1_ *k*_2_ *k*_3_], where the expression for *k*_*i*_ are given in Eq. 64. The Plücker line in each leg is orthogonal to vector **v**_*i*_ and passes through the centre of the revolute joint, as indicated by the red dashed lines in Figure 10.

### 3.9 3-FPR PCR

Each of the legs of the 3-FPR PCR contains a passive prismatic joint, indicating that the geometric constraint is the orthogonality between vectors **n**_*i*_ and **v**_*i*_ in the same leg

$$n_{i}^{T}v_{i}=0.$$ (66)

Before taking the time derivative of the constraint equations, we define vector **n**_*i*_, which is expressed in the fixed frame, in the following form

$$n_{i}=Q_{i1}Q_{i2}n_{i2},\;\;\;i=1,2,3$$ (67)

in which matrix **Q**_*i*1_ represents the constant orientation of the local frame *O*_*i*1_ *x*_*i*1_ *y*_*i*1_ with respect to the fixed frame, matrix **Q**_*i*2_ has the same form as that given in Eq. 4, and the constant unit vector **n**_*i*2_ is expressed in the local frame *O*_*i*2_ *x*_*i*2_ *y*_*i*2_. The time derivative of the constraint equations yields

$${\dot{n}}_{i}^{T}v_{i}+n_{i}^{T}{\dot{v}}_{i}=0.$$ (68)

Noting that both **Q**_*i*1_ and **n**_*i*2_ in Eq. 67 are constant with respect to time, as frame *O*_*i*1_ *x*_*i*1_ *y*_*i*1_ is fixed to the corresponding leg’s base and the direction of **n**_*i*2_ remains the same with respect to frame *O*_*i*2_ *x*_*i*2_ *y*_*i*2_, ${\dot{n}}_{i}$ can be written as

$${\dot{n}}_{i}=-{\dot{\kappa }}_{i}\ell_{i}Q_{i1}{EQ}_{i2}n_{i2}=-{\dot{\kappa }}_{i}\ell_{i}En_{i}.$$ (69)

Further, ${\dot{v}}_{i}$ is defined as

$${\dot{v}}_{i}=\dot{p}+\dot{\phi }{EQb}_{i0}-{\dot{u}}_{i}.$$ (70)

In this design **u**_*i*_ = **Q**_*i*1_ **u**_*i*1_, where vector **u**_*i*1_ is expressed in the local frame *O*_*i*1_ *x*_*i*1_ *y*_*i*1_ using Eq. 1, while its derivative is given in Eq. 2. Thus, we can further write

$${\dot{v}}_{i}=\dot{p}+\dot{\phi }{EQb}_{i0}-{\dot{\kappa }}_{i}Q_{i1}E_{i}u_{i1}.$$ (71)

Substituting Eq. 69 and Eq. 71 into Eq. 68 and rearranging terms in a similar way than for the previous design, one can obtain

$$n_{i}^{T}\dot{p}+\dot{\phi }n_{i}^{T}{EQb}_{i0}={\dot{\kappa }}_{i}k_{i},$$ (72)

in which the diagonal elements in Jacobian matrix **K** are

$$k_{i}=n_{i}^{T}\left(Q_{i1}E_{i}u_{i1}-\ell_{i}{Ev}_{i}\right),$$ (73)

and Jacobian matrix **J** is expressed as

$$J=\left[\begin{array}{cc} n_{1}^{T} & n_{1}^{T}{EQb}_{10}\\ n_{2}^{T} & n_{2}^{T}{EQb}_{20}\\ n_{3}^{T} & n_{3}^{T}{EQb}_{30} \end{array}\right].$$ (74)

**FIGURE 11:**
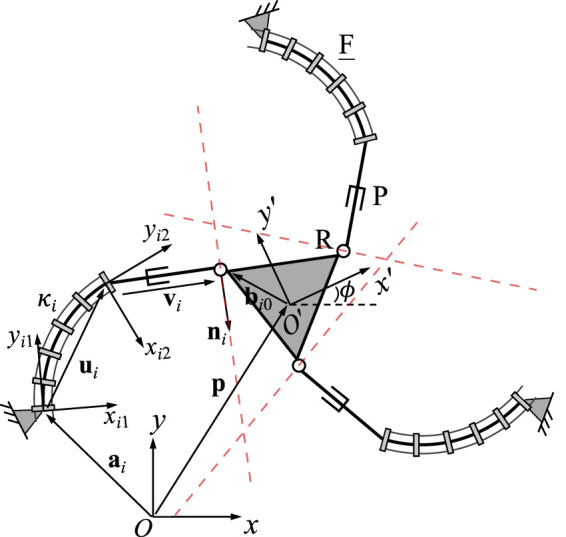
Geometric description of a planar three-DOF 3-FPR PCR.

The Plücker line in each leg is orthogonal to vector **v**_*i*_ and passes through the centre of the revolute joint, as indicated by the red dashed lines in Figure 11.

## 4 Singularity analysis

In this section, the previously derived Jacobian matrices **J** and **K** are used to provide a singularity analysis for each of the discussed PCR designs. In particular, we provide intuitive geometrical interpretations of the occurrences of type II (det(**J**) = 0) and type I singularities (det(**K**) = 0), while drawing comparisons to conventional 3-DOF PKM. Both type I and type II singularity loci of the PCRs can be obtained using a geometrical methods, comparable to what has been done in (Bonev et al., 2003) for conventional PKM. We believe that the presented results can guide future research endeavours concerned with planar 3-DOF PCR. For instance, knowledge about the singularity loci and occurrences can be used as a guidance not only for the choice and design of a non-redundant architecture for a particular task (Bonev et al., 2003), but also for synthesising redundant parallel manipulators to alleviate singularities (Wen et al., 2020).

### 4.1 Type II singularities

In (Bonev et al., 2003), the concept of reciprocal screws has been utilized to kinematically model planar PKMs. Although the velocity equations of the planar PCRs in this study are not developed based on this concept, the Jacobian matrices **J** have a similar physical meaning like the corresponding matrices in (Bonev et al., 2003). Therefore, type II singularity analysis of planar PCRs and their rigid counterparts can be carried out using the same framework, such as Grassmann line geometry (Wen and Burgner-Kahrs, 2022).

As pointed out in the previous section, each of the rows in Jacobian matrix **J** of a planar PCR can be represented by a Plücker line. Based on our findings, such a Plücker line can be geometrically identified by the following rules:

1) If a leg of the planar PCR contains two passive revolute joints, the Plücker line passes through the centres of both joints;
2) If a leg contains a passive revolute and a passive prismatic joint, the Plücker line passes through the centre of the former joint while being orthogonal to the axis of the latter joint.

**FIGURE 12:**
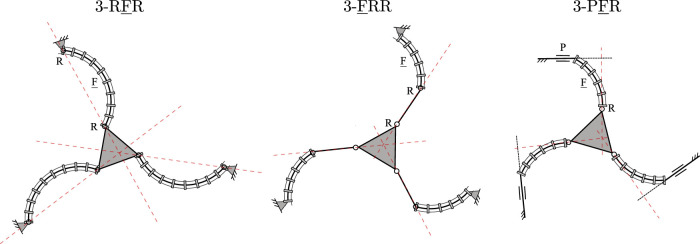
Example type II singularity occurrences for three of the considered PCR designs: 3-RFR, 3-FRR and 3-PFR.

Generally, Jacobian matrix **J** becomes singular and type II singularities occur when its three Plücker lines intersect at a common point or are parallel to each other. The loci and occurrences of these singularities depend on both the current joint configuration of the PCR, but also on its design parameters and the leg assembly. Example type II singularity occurrences for three of the considered 3-DOF PCR, namely the 3-RFR, 3-FRR and 3-PFR designs, are shown in Figure 12. The occurrences of II singularities for the other designs can be obtained analogously by investigating the corresponding Plücker lines of **J**.

It is noted, that the Plücker lines for the 3-RFP, 3-PFR, 3-PRF, and 3-FRP PCRs can never be parallel as long as the prismatic joints are not aligned. Due to this, some of these designs might in fact be free of type II singularities, if their Plücker lines additionally never intersect. For instance, type II singularities cannot occur in the 3-RFP and 3-FRP PCRs, since their Plücker lines will never intersect in praxis due to mechanical interferences.

It can be concluded that type II singularity analysis is very straightforward, as the Plücker lines can be identified in a geometrically intuitive way. It is further apparent that the loci and occurrences of type II singularities are mostly analogous to conventional PKM, which usually have Jacobian matrices **J** consisting of similar Plücker lines. However, throughout the next section we will show, that the type I singularity occurences for the PCR studied in this work are quite different from conventional PKM and are less intuitive to understand.

### 4.2 Type I singularities

**FIGURE 13:**
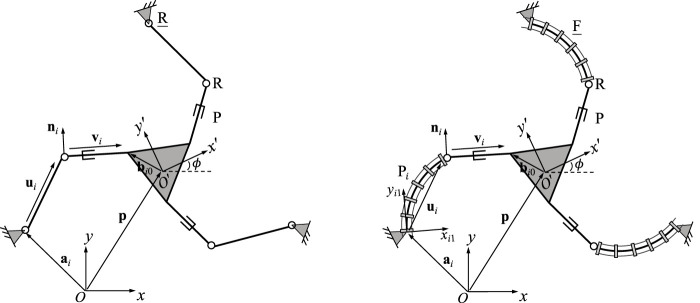
Geometric description of a planar three-DOF 3-RRP parallel mechanism (left) in comparison to the 3-FRP continuous counterpart (right).

Type I singularities of rigid PKMs generally occur when either an actuator (usually a linear actuator) reaches its extremes of extension or when the determinant of the Jacobian matrix **K** is equal to 0. While the first case is usually known directly from the resulting joint values of a design, the second case can often be identified in a straightforward manner by observing the configuration and resulting geometry of the manipulator. However, due to the bending motions of the continuum links, this geometric method might not be intuitive for PCRs. To elaborate on this difference, a comparison study of a rigid 3-RRP parallel architecture and a 3-FRP PCR is provided in the following. Both designs, which are exhibiting a similar kinematic structure, are depicted in Figure 13.

The Jacobian matrix **K** of the conventional 3-RRP robot can be expressed as

$$K=\left[\begin{array}{ccc} n_{1}^{T}{Eu}_{1} & 0 & 0\\ 0 & n_{2}^{T}{Eu}_{2} & 0\\ 0 & 0 & n_{3}^{T}{Eu}_{3} \end{array}\right],$$ (75)

where **E** is a 90-degree rotation as defined in Eq. 6. The diagonal entries of the corresponding matrix for the continuous counterpart, i.e., the 3-FRP robot, are given in Eq. 50, such that matrix **K** results in

$$K=\left[\begin{array}{ccc} n_{1}^{T}Q_{11}E_{1}u_{11} & 0 & 0\\ 0 & n_{2}^{T}Q_{21}E_{2}u_{21} & 0\\ 0 & 0 & n_{3}^{T}Q_{31}E_{3}u_{31} \end{array}\right],$$ (76)

where **Q**_*i*1_ is the orientation of the base of continuum link in leg *i*, **E**_*i*_ is defined in Eq. 3 and **u**_*i*1_ is vector **u**_*i*_ expressed in the local frame *O*_*i*1_ *x*_*i*1_ *y*_*i*1_ as defined in Eq. 1.

It can be observed from Eq. 75 that a type I singularity occurs when vectors **n**_*i*_ and **u**_*i*_ (with *i* = 1, 2, 3) are aligned, i.e., the distal link is perpendicular to the proximal link in the same leg (Bonev et al., 2003), resulting in det(**K**) = 0. Thus the occurrences of type I singularities can always be associated with the orientation of these two links, allowing to intuitively detect them based on geometry.

On the contrary, the determinant of Jacobian matrix **K** for the 3-FRP PCR becomes 0 when vector **n**_*i*_ is orthogonal to vector **Q**_*i*1_ **E**_*i*_ **u**_*i*1_ (with *i* = 1, 2, 3). However, unlike for its rigid counterpart, the direction of the latter vector, which is different from vector **Eu**_*i*_ in the 3-RRP rigid parallel mechanism, is not constantly associated with a link. In fact, its direction is always tangent to the course of the tip of the corresponding continuum link, which is visualized in Figure 1. Thus, the orthogonality between these two vectors is not intuitively observed from the configurations of the PCR.

Throughout the remainder of this section, we are providing additional insights into the occurrence of type I singularities for each PCR design discussed in this paper. We are further investigating the existence of different possible postures of the legs in each design for a given desired end-effector pose. This concept has first been discussed for conventional planar parallel robots by Chablat and Wenger (1998), who further introduce the idea of working modes that are separating sets of different leg postures. We show, that type I singularities of the PCR discussed in this paper separate different leg posture solutions. Thus, type I singularities occur, when the number of possible leg posture solution varies, e.g., when the number of possible solutions changes from two to one or from four to two. In this case, two separate working modes of the PCR join. We note that an analogous behaviour was shown for conventional PKM by Chablat and Wenger (1998) and was conjectured for passive link PCR by Briot and Goldsztejn (2021).

**FIGURE 14:**
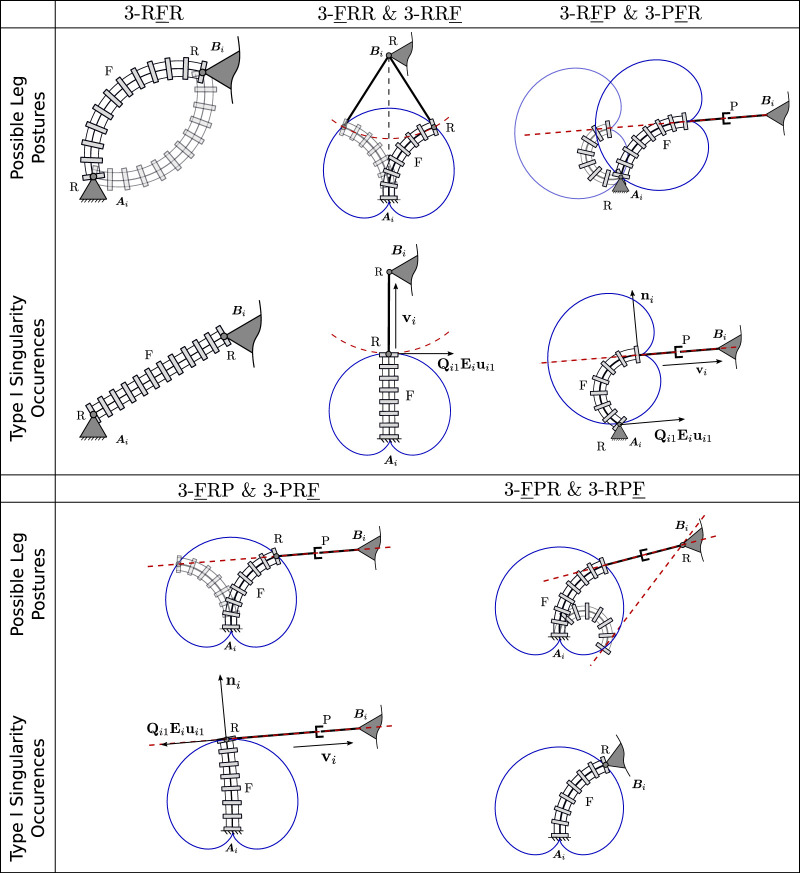
Visualization of different leg postures (top) and example type I singularity occurrence (bottom) for one leg in each discussed PCR design. The course of the continuum links distal end is visualized in a solid blue line, while the possible motions of the passive link in each leg is shown using a dashed red line.

The results of this section are depicted in Figure 14. For each design, we show possible multiple leg postures for one leg of the corresponding design, connecting the leg’s base *A*_*i*_ to the platform coupling point *B*_*i*_. The shown solutions meet the geometrical coupling constraints, which depend on possible positions of the end of the continuum link, shown in a blue solid line, and the possible motions allowed from the passive joint, shown in red dashed line. The figure further shows example cases, in which type I singularities for an individual leg in a design exist. As stated above, the number of possible leg postures changes in these configurations. Additionally, the tangents of the dashed red line and solid blue line at the corresponding solution become parallel for most designs in this case. We believe that the insights gained in this section can lead to a better geometrical understanding of type I singularity occurrences, which can for instance be useful to determine the designs workspace boundaries, which are usually defined by such.

We note that observing the results from the previous section, it can be seen that designs that are featuring the same kinematic structure in their legs, but with a reversed arrangement, have similar type I singularity conditions. For instance, the 3-FRR and 3-RRF designs have type I singularities when Eq. 18 and Eq. 26 are equal to 0 for any *i*. It can be seen that these equations only differ in their sign, leading to the same conditions for type I singularities. The same is true for most of the remaining designs, with the 3-RFR being the exception. Thus, through the following we will only discuss one of each of these designs, while the type I singularity occurrences of the corresponding other design can be obtained analogously.

#### 4.2.1 3-RFR PCR

For the 3-RFR PCR, generally two configurations for each leg exist for a given end-effector pose. In both solutions, the continuum link exhibits the same magnitude of curvature *κ*_*i*_, however is bent in different directions. This is analogous to the typical elbow-up and elbow-down solutions for conventional planar parallel robots. When the number of inverse kinematic solutions reduces to one, a type I singularity occurs. This is the case for *κ*_*i*_ = 0, resulting in a straight continuum link. Further, the matrix **K** for this specific PCR design in Eq. 12 becomes singular in this case, confirming the existence of a type I singularity. In addition, type I singularities occur, when $|\kappa_{i}|=2\pi \ell_{i}^{-1}$ , which is the maximum bending curvature, in which the continuum link forms a closed circle. In this case, vector **u**_*i*_ = **0**, and *k*_*i*_ = 0. In fact, this leads to both a type I and type II singularity, as both **J**, as defined in (10), and **K**, as defined in Eq. 12, become singular.

#### 4.2.2 3-FRR and 3-RRF PCR

For the 3-FRR PCR, the different configurations for each leg, given the pose of the end-effector, can be identified by investigating the possible positions of the continuum link and the possible motions allowed by the passive joints. Both are depicted in Figure 14 using a blue solid line and a red dashed line respectively. Valid leg configurations exist at intersections of these two lines. In the shown case, two intersections and thus configurations exist, in which the continuum link exhibits different bending curvatures *κ*_*i*_. However, depending on the occurring intersections between the red and blue lines, up to four different solutions can be identified, if the distance between *B*_*i*_ and *A*_*i*_ decreases.

When the number of intersections and possible leg configurations changes, i.e., in the example the number of solutions reduces from two to one, a type I singularity occurs. In this case, **Q**_*i*1_ **E**_*i*_ **u**_*i*1_, which is tangent to the trajectory of the continuum link (blue), is orthogonal to **v**_*i*_, which is parallel to the rigid link in the kinematic chain. In other words, the tangent of the blue line **Q**_*i*1_ **E**_*i*_ **u**_*i*1_ becomes parallel to the red dashed line. The singularity occurrence can be confirmed by investigating the corresponding matrix **K** for this specific PCR design, which entries are defined by Eq. 18, as it becomes singular in this configuration. The same phenomena can be observed in other instances, e.g., when the number of solutions reduces from four to two. Additionally, similar to the previous design, a type I singularities also occurs for the maximum bending curvature $|\kappa_{i}|=2\pi \ell_{i}^{-1}$ . In this case, **u**_*i*_ = **0**, leading to a singularity in **K**.

These observed results are equivalent for the 3-RRF PCR, as the individual entries of **K** in Eq. 26 result in a similar expression compared to Eq. 18.

#### 4.2.3 3-RFP and 3-PFR PCR

The possible leg configurations for the 3-RFP PCR can again be obtained by investigating the possible positions of the continuum link in blue and the possible motions allowed by the passive joints in red. For a given end-effector pose the position of point *B*_*i*_ is known, thus, the distal end of the continuum link is constrained to lie on the red dashed line. Further, the proximal end of the continuum link, whose possible positions are denoted by the solid blue line, has to align with point *A*_*i*_. From this, exactly two leg configurations can be identified using the sketch in Figure 14 for the shown case. The main difference between these solutions is the extension of the passive prismatic joint and the bending curvature *κ*_*i*_ of the continuum link. We note that the number of possible solutions in the general case could differ from two, as it depends on the position of *B*_*i*_ with respect to *A*_*i*_ and the fixed orientation between the prismatic joint and the continuum link.

In the case, in which the number of solutions reduces, e.g., in our example only one possible intersection between the proximal end of the continuum link and point *A*_*i*_ exists, a type I singularity occurs. In this case, **Q**_*i*1_ **E**_*i*_ **u**_*i*1_, which is tangent to the trajectory of the continuum link (blue), is orthogonal to **n**_*i*_, which itself is orthogonal to the rigid link in the kinematic chain. In other words, the tangent of the blue line **Q**_*i*1_ **E**_*i*_ **u**_*i*1_ becomes parallel to the red dashed line. This can be confirmed by investigating the corresponding matrix **K** for this specific PCR design, which entries are defined by Eq. 35, as it becomes singular in this configuration. Again, a type I singularities also occurs for the maximum bending curvature $|\kappa_{i}|=2\pi \ell_{i}^{-1}$ . In this case, **u**_*i*_ = **0**, leading to a singularity in **K**. Another straightforward type I singularity occurs, when the prismatic joint is fully retracted such that **v**_*i*_ = **0**. In this case, vector **n**_*i*_ becomes undefined. This additionally leads to a type II singularity, since **J**, as defined in Eq. 34, exhibits a zero-row in this case. However, in praxis, this PCR configuration might not be attainable due to mechanical limits and interferences.

These observed results are equivalent for the 3-PFR PCR, as the individual entries of **K** in Eq. 41 result in a similar expression compared to Eq. 35.

#### 4.2.4 3-FRP and 3-PRF PCR

The leg configurations of the 3-FRP PCR are obtained analogous to the previous designs. The red dashed line denotes the possible positions defined by the passive prismatic joint. The different leg configurations exist, where this line intersects with the possible positions of the distal end of the continuum segment shown in blue. The shown example shows two possible solutions, where both feature different bending curvatures *κ*_*i*_ of the continuum link. In the general case, more than just two solutions might exists, depending on the number of intersections of the lines.

In the case that the number of solution changes, e.g., in the shown example only one intersection between the two lines exists, a type I singularity occurs. Here, similar to the previous design, **Q**_*i*1_ **E**_*i*_ **u**_*i*1_, which is tangent to the trajectory of the continuum link (blue), is orthogonal to **n**_*i*_, which itself is orthogonal to the rigid link in the kinematic chain. Thus, the tangent of the blue line **Q**_*i*1_ **E**_*i*_ **u**_*i*1_ becomes parallel to the red dashed line. Again, we can confirm the existence of a type I singularity by investigating the corresponding matrix **K** for this design, which entries are defined by Eq. 50, which becomes singular in this configuration. Again, type I singularities also occur for $|\kappa_{i}|=2\pi \ell_{i}^{-1}$ , leading to **u**_*i*_ = **0**, and for **v**_*i*_ = **0**, in which case **n**_*i*_ becomes undefined. The latter case also leads to a type II singularity.

These observed results are equivalent for the 3-PRF PCR, as the individual entries of **K** in Eq. 45 result in a similar expression compared to Eq. 50.

#### 4.2.5 3-FPR and 3-RPF PCR

For the 3-FPR design, the possible leg configurations for a given end-effector pose are not as intuitive as for the previous designs. In order to identify them, we can visualize the possible positions of the continuum link’s distal end in blue. Considering, that the passive prismatic joint is rigidly attached to this end with a given orientation, we can draw the possible extension along this link with a dashed red line. A valid leg configuration is found, if for a given continuum link position, this red line intersects with point *B*_*i*_. Two possible leg configurations are visualized in Figure 14 as an example. However, we note that in praxis, one of these solutions would be unattainable due mechanical limits and interferences.

Geometrically identifying type I singularities, i.e., configurations, in which only one leg configuration exists for a given end-effector pose, is challenging for the 3-FPR PCR design. When observing the individual entries of the **K** matrix for this design in Eq. 73, it can be seen that a type I singularity exists, when vector **n**_*i*_ is orthogonal to vector (**Q**_*i*1_ **E**_*i*_ **u**_*i*1_−*ℓ*_*i*_ **Ev**_*i*_). The latter vector consists of a vector, that is, tangent to the continuum link’s distal end’s trajectory and a vector, that is, orthogonal to **v**_*i*_ and parallel to **n**_*i*_. Due to this, the vector (**Q**_*i*1_ **E**_*i*_ **u**_*i*1_−*ℓ*_*i*_ **Ev**_*i*_) will never be orthogonal to vector **n**_*i*_. The only cases, in which type I singularities occur, is when either vector **v**_*i*_ = **0** and vector **n**_*i*_ becomes undefined, or when **Q**_*i*1_ **E**_*i*_ **u**_*i*1_ = *ℓ*_*i*_ **Ev**_*i*_, such that the second vector becomes a null-vector. Thus, unlike for the other designs, a type I singularity does not occur in the maximum bending curvature $|\kappa_{i}|=2\pi \ell_{i}^{-1}$ .

These observed results are analogous for the 3-RPF PCR, however when observing Eq. 64, we can see that **Q**_*i*1_ **E**_*i*_ **u**_*i*1_ = −*ℓ*_*i*_ **Ev**_*i*_ for the second type I singularity condition.

## 5 Limitations

We would like to acknowledge, that while nine possible PCR designs have been presented throughout this work, not all of these outlined designs might be physically feasible, depending on the chosen actuation method. For instance, tendon-driven continuum links need a motor at either one of their ends to pull and release the routed tendons. Thus, this motor would need to move along the motion of potential passive revolute and prismatic joints, which might be difficult to realize in a robotic prototype or add significant weight to the resulting manipulator. We further note that the derivations throughout this work are only valid for PCR designs featuring continuum links bending in constant curvature arcs. This assumption is generally only true for certain actuation methodologies in the absence of external or coupling forces, potentially requiring additional passive joints to eliminate the latter according to Grübler’s formula. Lastly, the presented derivations only consider kinematic and geometric relations, while the statics of the manipulator are not modelled. Due to this, not all robot configurations that fulfill the geometric constraints might result in a static equilibrium, which would have to be evaluated with respect to external loads and internal wrenches exchanged by the robot links and the end-effector platform. In addition, instabilities or uncontrollable end-effector motions arising from the compliant material of the continuum links can not be considered within this framework.

## 6 Conclusion

In this work, we studied 3-DOF planar PCR designs, each consisting of three individual legs. Each leg features an actuated constant curvature continuum link in addition to two discrete passive joints. We showed that following this design paradigm and assuming symmetrical designs, 12 different kinematic leg structures exist, nine of them are suitable for composing 3-DOF planar PCRs. The velocity kinematics were derived for each of these designs based on their individual constraint equations. Based on these derivations a singularity analysis was conducted. It could be shown, that the type II singularity conditions of the proposed PCRs are the same as their rigid counterparts and can be determined in an intuitive geometric manner using Plücker lines. As this type of singularities only depends on the resulting geometry of each configuration, it is independent of the framework used to model the continuum link. Different from rigid 3-DOF planar parallel manipulators, the type I singularity conditions of the proposed PCRs are not as easily and intuitively observed geometrically. To alleviate this, we provided additional insights into the occurrences of these singularities, particularly connecting them to the existence of multiple possible leg configurations.

In conclusion, our work extends the current state of the art on the design and evaluation of planar 3-DOF PCR, which are using actuated continuum links bending in constant curvatures. We envision that our work can guide researchers to choose a specific 3-DOF PCR designs given particular applications and requirements. We also believe that our investigations can be helpful to further evaluate the kinematic properties with respect to the design parameters of a particular manipulator. For instance, the presented kinematic framework can be used to investigate the singularity distributions for each design as well as their dependence on different design parameters and leg configurations. Lastly, the velocity equations provided for each PCR design can be useful for controlling the manipulators under constant curvature assumption. We anticipate that our work can be extended to spatial PCR featuring similar individual legs with actuated constant curvature continuum links, such as the designs in (Garcia et al., 2020) and (Garcia et al., 2021), in a straightforward manner.

## Data Availability

The original contributions presented in the study are included in the article/Supplementary Material, further inquiries can be directed to the corresponding author.
